# Engaging Older Adult Learners Through Intergenerational Learning Opportunities

**DOI:** 10.1093/geroni/igaa057.1815

**Published:** 2020-12-16

**Authors:** Skye Leedahl, Phillip Clark, Beth Leconte

**Affiliations:** 1 University of Rhode Island, Kingston, Rhode Island, United States; 2 University of Rhode Island Osher Lifelong Learning Institute, Kingston, Rhode Island, United States

## Abstract

The University of Rhode Island became a part of the AFU network in 2018, and much of our rationale for joining the network was based on our strengths and growing interest in intergenerational programs and learning. The URI Osher Lifelong Learning Institute (OLLI) currently has over 1,300 members, and a large aspect of their strategic plan and current efforts are focused on increasing intergenerational learning opportunities due to interest and successes in these areas. Some of the successful strategies that have been used include an intergenerational classroom of OLLI member and university students designed within a traditional college class, a matching program where students are partnered with OLLI members, intergenerational service learning opportunities for students to engage with OLLI members in different ways, and question and answer sessions with OLLI members within college classes. This presentation will highlight these efforts, lessons learned, and efforts to track participation and outcomes.

